# Agreement between older adult patient and caregiver proxy symptom reports

**DOI:** 10.1186/s41687-022-00457-8

**Published:** 2022-05-14

**Authors:** Kurt Kroenke, Timothy E. Stump, Patrick O. Monahan

**Affiliations:** 1 Indiana University School of Medicine, Indianapolis, IN USA; 2 Regenstrief Institute, Inc, 1101 West 10th St, Indianapolis, IN 46202 USA; 3 Indiana University Fairbanks School of Public Health, Indianapolis, IN USA

**Keywords:** Depression, Anxiety, Pain, Symptoms, Psychometrics, Proxy report, Concordance

## Abstract

**Background:**

Proxy report is essential for patients unable to complete patient-reported outcome (PRO) measures themselves and potentially beneficial when the caregiver perspective can complement patient report. In this study, we examine agreement between self-report by older adults and proxy report by their caregivers when completing PROs for pain, anxiety, depression, and other symptoms/impairments.

**Methods:**

Four PROs were administered by telephone to older adults and their caregivers followed by re-administration within 24 h in a random subgroup. The PROs included the PHQ-9 depression, GAD-7 anxiety, PEG pain, and SymTrak multi-dimensional symptom and functional status scales.

**Results:**

The sample consisted of 576 older adult and caregiver participants (188 patient-caregiver dyads, 200 patients without identified caregiver). The four measures had good internal (Cronbach’s alpha, 0.76 to 0.92) and test–retest (ICC, 0.63 to 0.92) reliability whether completed by patients or caregivers. Total score and item-level means were relatively similar for both patient and caregiver reports. Agreement for total score as measured by intraclass correlation coefficient (ICC) was better for SymTrak-23 (0.48) and pain (0.58) than for anxiety (0.28) and depression (0.25). Multinomial modeling showed higher (worse) patient-reported scale scores were associated with caregiver underreporting, whereas higher caregiver task difficulty was associated with overreporting.

**Conclusion:**

When averaged over individuals at the group level, proxy reports of PRO scores by caregivers tend to approximate patient reports. For individual patients, proxy report should be interpreted more cautiously for psychological symptoms as well as when patient-reported symptoms are more severe, or caregiver task difficulty is high.

## Plain English summary

Assessment of patient-reported outcomes (e.g., symptoms, functional status, and other quality of life domains) by a proxy on behalf of the patient is helpful when patient self-report is not possible or when a complementary perspective may inform care. Determining how well older adult patients and their caregivers agree on the patient’s pain, depression, anxiety, and functioning is important in investigating and managing these core clinical domains. In this study, patient-caregiver agreement was evaluated for four commonly-used scales that assess depression (PHQ-9), anxiety (GAD-7), pain (PEG), and multi-dimensional symptom and functional impairment (SymTrak). Total score and item-level scores were similar between patients and caregivers when averaged at the group level. Agreement was higher for more physically oriented domains (pain and SymTrak) than psychological conditions (depression and anxiety). Higher (worse) patient-reported scale scores were associated with caregiver underreporting, whereas higher caregiver task difficulty was associated with overreporting. Proxy report may be sufficiently accurate in research when studying group differences. However, proxy reports should be interpreted more cautiously in individual patients with psychological symptoms or higher symptom severity, or where there is caregiver diffficulty.

## Introduction

Patient-reported outcome (PRO) measures are increasingly being used to assess symptoms and functioning that rely heavily on patient input to rate the presence and severity of problems with these domains [1–4]. PROs typically require persons to have the cognitive and communicative capacity to assess what they are experiencing and communicate this to others. There are situations, however, where assessment by a caregiver or other proxy may either be necessary (e.g.., substituted ratings in patients with substantial cognitive impairment) or of added benefit (e.g., if the proxy reporter has a different perspective that complements ratings provided by the patient) [5–8]. This raises the essential question of how accurately a proxy can evaluate a domain that is being experienced solely or predominantly by the patient. This is important in order to decide if and when clinicians should gather proxy report and ultimately act upon the information.

Pain, anxiety, and depression (the PAD triad) are among the most common and disabling symptoms in the general and clinical population and are frequently under-recognized and suboptimally treated [9]. Moreover, they commonly co-occur, adversely affect treatment responsiveness of one another, and are responsible for an enormous amount of disability as well as direct and indirect medical and societal costs [8]. The PHQ-9 depression, GAD-7 anxiety, and PEG pain scales are among the most widely-used PROs for assessing PAD symptoms in both research and clinical practice [10–12]. Moreover, the PHQ-9 and GAD-7 have been recommended as core outcome measures in older adults [13]. Additionally, aging is often accompanied by multimorbidity, i.e., the co-occurrence of several diseases in the same individual. To address this issue, SymTrak has recently been developed and validated as a multi-dimensional scale that measures common symptoms and impairments in older adults [14, 15].

In this paper, we analyze PRO data from older adults and caregivers who participated in a cohort study to develop the SymTrak scale. Specifically, we focus on Symtrak and PAD scale scores as the PROs of interest. Our objectives are to:

1. Assess the internal consistency and test–retest reliability of patient and caregiver PRO report;
2. Determine item-level and scale-level agreement (concordance) between patient and caregiver PRO report;
3. Examine the association between patient-caregiver concordance and symptom severity, while adjusting for potentially confounding covariates.

## Methods

### Study participants

A group of 576 participants (188 patient-caregiver dyads and 200 non-dyadic patients without an identified caregiver) recruited from an academic-affiliated primary care network of clinics constitute the sample for this study. Patient inclusion criteria were: (1) age ≥ 65 years, (2) ≥ one primary care visit in the past 12 months, (3) ≥ one chronic condition according to medical records, and (4) for those participants with an informal caregiver, the caregiver had to be ≥ 21 years of age and willing to participate in the study. Patients with a serious mental illness such as bipolar disorder or schizophrenia or who resided in a long-term care facility were excluded. The study was approved by the Indiana University Institutional Review Board (IRB #1308983443), and all participants provided written informed consent. Further details of study procedures is described elsewhere [14, 15].

### Measures

Participants completed by telephone interview a brief survey which included the scales assessed in this study. A random third of the patients and caregivers were re-interviewed 24 h later to assess test-test reliability. Caregiver and patient versions of the scales had identical item wording and response options except that “your loved one” was substituted for “you” in the caregiver version items to ensure proxies were reporting from the patient’s perspective.

SymTrak is a 23-item multi-morbidity scale that focuses on clinically actionable symptoms and impairments common in older adults. Response options for each item are: 0 = Never, 1 =  Sometimes, 2 = Often, 3 = Always. Thus, SymTrak scores from 0 to 69. The construct and factorial validity as well as sensitivity to change of Symtrak has been demonstrated [14, 15].

The PEG is a 3-item pain scale that assesses average pain intensity as well as pain interference with enjoyment of life and general activities in the past week. Each item is scored on 0 to 10 scale, with the PEG score being the mean of the 3 items and higher scores representing worse pain. Validity and responsiveness of the PEG is comparable to longer legacy pain measures [16, 17].

The Patient Health Questionnaire 9-item depression scale (PHQ-9) is one of the most widely-used measures for assessing depression in both clinical practice and research [10, 12]. In this study, the PHQ-8 was used which is identical to the PHQ-9 except it omits the 9^th^ item which assesses suicidal ideation. The PHQ-8 is often used in clinical research settings where depression is not the primary outcome and endorsement of the 9^th^ item is most often a false positive response for active suicidal ideation. Because the 9^th^ item is the least frequently endorsed item, multiple studies have shown that group mean scores are nearly identical for the PHQ-8 and PHQ-9 as is the optimal screening cutpoint of ≥ 10 [18].

The Generalized Anxiety Disorder 7-item anxiety scale (GAD-7) is a measure for anxiety screening and severity assessment [10]. Although initially developed as a measure for generalized anxiety disorder, the GAD-7 also has good operating characteristics as a screener for panic disorder, social anxiety disorder, and posttraumatic stress disorder [19]. It is one of the most widely-used brief anxiety measures [20].

The difficulty subscale from the Oberst Caregiver Burden Scale was used to measure caregiver perceptions of difficulty for 15 different types of caregiving tasks [21]. Each of the 15 Items are rated on a 5-point scale ranging from 1 (*not difficult)* to 5 (*extremely difficult*). Thus, scores range from 15 to 75 with higher scores representing greater caregiver difficulty.

### Analysis

Cronbach’s alpha was used to assess internal consistency reliability. The intra-class correlation coefficient (ICC) was used for two types of analyses. The absolute-agreement version of the ICC was used to assess test–retest reliability for scale scores, with occasions specified as a random effect. Agreement between patients and caregivers on scale scores was assessed using the absolute-agreement ICC, with a fixed effect for rater (patient, caregiver). Cronbach’s alpha and ICC are considered acceptable if ≥ 0.70. Agreement on patient-caregiver item-level ordinal responses was assessed with the weighted kappa (using Fleiss-Cohen quadratic weights) as the primary statistic and the Spearman correlation coefficient as a secondary index. Quadratic weights, which are commonly used for weighted kappa, were implemented [22]. Moreover, the formula for weighted kappa when using quadratic weights is nearly identical to the formula for an ICC specified above [23]. However, as a sensitivity analysis, we also calculated weighted kappas using linear weights. Agreement was considered substantial if kappa was > 0.61 to 0.80, moderate if 0.41 to 0.60, fair if 0.21 to 0.40, and slight if 0.01 to 0.20 [24]. Scatter plots of patient versus caregiver total scores were used to examine whether agreement varied with symptom severity.

Measurement error bars were based upon the standard error of measurement (SEM) which was calculated as ${\text{SD}} \times \sqrt {1 - \alpha }$, where α = Cronbach’s alpha. Error bars were set at ± 2 SEM since differences larger than this are considered by some to represent minimally important differences [25]. Multinomial logistic regression modeling was done to explore patient and caregiver characteristics associated with caregiver overreporting and underreporting (i.e., caregiver-reported score > 2 SEM higher or lower than patient-reported score, respectively). Odds ratios (ORs) and 95% confidence intervals were reported.

## Results

### Participant characteristics

*Dyads.* Patients and caregivers were diverse with respect to race, education, income, and marital status (Table 1). Of the 203 recruited patient-caregiver dyads, some patients or caregivers subsequently decided to not participate, yielding 188 dyads available for concordance analyses. Most caregivers were either a child (42%) or a spouse/partner (37%) of the dyadic patients (Table 1). The mean (SD) Oberst caregiver task difficulty score was 20.7 (8.6) with a range of 15 to 65.

**Table 1:** Characteristics of patient and caregiver dyads

Characteristic	Patients (n = 188)	Caregivers (n = 188)
Age, mean (SD);	75.3 (7.0)	59.3 (12.9)
*Age categories, No. (%)*		
< 50	0 (0.0)	44 (23.9)
50–64	0 (0.0)	69 (37.5)
65–74	97 (52.7)	54 (29.4)
> = 75	87 (47.3)	17 (9.2)
Female sex, No. (%)	130 (69.2)	140 (74.5)
*Race, No. (%)*		
White	93 (49.5)	94 (50.0)
Black	89 (47.3)	90 (47.9)
Other	6 (3.2)	4 (2.1)
*Highest level of education, No. (%)*		
< High school graduate	65 (34.6)	29 (15.4)
High school graduate	45 (23.9)	63 (33.5)
Some college or higher	78 (41.5)	96 (51.1)
*Total household income, past year, No. (%)*		
< $15,000	77 (41.0)	54 (28.7)
$15,000-$30,000	49 (26.1)	51 (27.1)
> $30,000	51 (27.1)	68 (36.2)
Unknown	11 (5.9)	15 (8.0)
*Marital status, No. (%)*		
Married/living together	72 (38.3)	102 (54.3)
Widowed	67 (35.6)	12 (6.4)
Divorced/separated	35 (18.6)	36 (19.2)
Never married	13 (6.9)	38 (20.2)
Unknown	1 (0.5)	0 (0.0)
*Relationship to Patient, No. (%)*		
Spouse or partner		70 (37.2)
Child		79 (42.0)
Sibling		15 (8.0)
Grandchild		4 (2.1)
Parent		2 (1.1)
Other		18 (9.6)

*Non-dyadic patients.* Patients without a participating caregiver were included in scale reliability analyses and, compared to dyadic patients, were significantly younger by an average of 2 years (p = 0.01), less likely to be married or living with a partner (p < 0.0001) and with lower household income (p < 0.0001).

### Reliability

Internal consistency reliability was high for all four scales and comparable across non-dyadic and dyadic patients as well as caregivers (Table 2). Of the 16 Cronbach’s alpha calculations, 13 ranged from 0.83 to 0.94. The remaining three ranged from 0.75 to 0.78 and all related to depression.

**Table 2:** Internal Consistency and Test–Retest Reliability of the Four Scales

Scales	Internal consistency reliability (Cronbach’s alpha)	Test–retest reliability (Intra-class correlation)
Patients, total	(n = 388)	(n = 121)
SymTrak	0.85	0.88
PHQ-8 depression	0.76	0.73
GAD-7 anxiety	0.84	0.80
PEG pain	0.90	0.87
Patients, non-dyadic	(n = 200)	(n = 62)
SymTrak	0.85	0.87
PHQ-8 depression	0.75	0.83
GAD-7 anxiety	0.83	0.85
PEG pain	0.89	0.81
Patients, dyadic	(n = 188)	(n = 59)
SymTrak	0.86	0.88
PHQ-8 depression	0.78	0.63
GAD-7 anxiety	0.85	0.74
PEG pain	0.92	0.92
Caregivers, dyadic	(n = 188)	(n = 65)
SymTrak	0.87	0.91
PHQ-8 depression	0.83	0.92
GAD-7 anxiety	0.87	0.74
PEG pain	0.94	0.96

Test–retest reliability also revealed high agreement for all scales and was generally similar across patients and caregivers (Table 2). Of the 16 test–retest calculations, 12 were 0.80 or greater, 3 were 0.73 to 0.74, and one was 0.63. The four test–retest results less than 0.80 were either depression (n = 2) or anxiety (n = 2).

### Concordance for SymTrak

As shown in Table 3, the SymTrak mean total scores were similar for patient-reported and caregiver-reported proxy scores (17.5 vs. 18.1). Concordance for the total score was in the poor to moderate range (ICC = 0.48; Spearman’s correlation = 0.49). Item mean scores were quite similar with no patient-proxy item mean differing by greater than 0.2. In addition, 18 of the 23 items showed either moderate (n = 8) or fair (n = 10) patient-caregiver concordance as reflected by a weighted (quadratic) kappa of ≥ 0.40 and ≥ 0.20, respectively. Spearman correlation results generally paralleled weighted kappa findings. Linear weighted kappas were typically somewhat lower than quadratic weighted kappas. Of the five items with poor concordance, 2 were psychological (items 14 and 19), 2 were cognitive (items 20 and 22), and 1 was trouble with urination.

**Table 3:** Patient-Caregiver Concordance for SymTrak Scale

Item #^a^	SymTrak Items	Dyads	Item mean (SD)		Spearman Correlation^b^	Weighted Kappa^c^	
		N	Patients	Caregivers		Quadratic^d^	Linear
	*Moderate agreement*						
11	Trouble with hearing	186	0.9 (1.0)	0.8 (1.1)	0.54	0.56	0.39
4	Pain in the back, arms, legs, or joints	187	1.6 (1.1)	1.5 (1.1)	0.54	0.53	0.40
8	Shortness of breath	187	0.8 (0.9)	0.8 (0.9)	0.53	0.53	0.43
12	Trouble walking or trouble moving around	186	1.2 (1.0)	1.3 (1.1)	0.51	0.49	0.39
10	Trouble with vision	187	0.9 (1.0)	0.8 (1.0)	0.40	0.46	0.32
3	Pain interfering with daily activities	187	1.2 (1.1)	1.3 (1.0)	0.44	0.42	0.30
6	Constipation or stomach problems	186	0.7 (0.8)	0.8 (0.8)	0.39	0.41	0.31
9	Chest pain	187	0.3 (0.6)	0.3 (0.6)	0.45	0.41	0.39
	*Fair agreement*						
1	Feeling tired or having low energy	187	1.3 (0.9)	1.4 (0.9)	0.37	0.35	0.27
5	Foot pain or foot numbness	186	1.0 (1.0)	0.8 (1.0)	0.37	0.34	0.26
16	Poor appetite or overeating	187	0.7 (0.8)	0.7 (0.9)	0.34	0.33	0.24
2	Trouble falling asleep or trouble staying asleep	187	1.0 (0.9)	0.9 (0.9)	0.29	0.30	0.21
15	Feeling sad, down, or depressed	187	0.5 (0.7)	0.7 (0.8)	0.35	0.29	0.26
17	Feeling nervous or anxious	187	0.6 (0.7)	0.7 (0.8)	0.22	0.29	0.20
23	Memory loss	187	0.7 (0.7)	0.7 (0.8)	0.27	0.28	0.21
18	Worrying too much about different things	188	0.8 (0.9)	1.0 (1.0)	0.26	0.25	0.20
21	Trouble remembering appointments	187	0.4 (0.6)	0.6 (0.8)	0.21	0.25	0.16
13	Falling or tripping	186	0.3 (0.5)	0.4 (0.6)	0.29	0.24	0.24
	*Slight agreement*						
19	Becoming easily annoyed or irritable	186	0.6 (0.7)	0.8 (0.8)	0.18	0.18	0.14
14	Less interest or less pleasure in doing things	187	0.7 (0.8)	0.8 (0.9)	0.16	0.17	0.08
22	Trouble concentrating on things	187	0.6 (0.7)	0.6 (0.7)	0.13	0.16	0.09
7	Trouble with urination	185	0.5 (0.8)	0.5 (0.8)	0.13	0.07	0.08
20	Trouble taking medications in the right dose at the right time	187	0.3 (0.6)	0.4 (0.6)	0.13	0.07	0.09
	SymTrak scale score^d^	188	17.5 (9.5)	18.1 (10.1)	0.49	0.48 (0.38–0.57)	

^**a**^Items are ordered in table from highest to lowest quadratic weighted kappa

^**b**^Most Spearman correlations are highly significant (p < .0001) except for items only significant at p < .05 (items 14, 17, 18, 19, 21) or those not significant (items 7, 20, 22)

^**c**^All weighted kappa values are significant (i.e., 95% CI does not include 0) except for items 7 and 20 (quadratic and linear), as well as items 14 and 22 (linear only)

^**d**^For SymTrak scale score, the value under the weighted kappa column is the Intraclass Correlation Coefficient (ICC) with 95% CI, calculated using the absolute agreement version of the ICC and specifying rater (patient vs their caregiver) as a fixed effect

### Concordance for pain, anxiety and depression

Table 4 summarizes patient-proxy agreement for PEG pain, GAD-7 anxiety, and PHQ-8 depression scores. In general, concordance was higher for pain than the two psychological scores. PEG total and item mean scores were similar between patients and caregivers, and both the ICC and Spearman’s correlation were 0.58.

**Table 4:** Patient-Caregiver Concordance for Pain, Anxiety, and Depression Scales

Item #^a^	Item	Item mean (SD)		Spearman correlation^b^	Weighted Kappa^c^	
		Patients	Care-givers		Quadratic	Linear
	*PEG pain*					
1	Pain on average in the past week	4.7 (3.1)	4.8 (3.1)	0.57	0.57	0.43
2	Pain interference with enjoyment of life	3.8 (3.5)	4.1 (3.5)	0.50	0.50	0.37
3	Pain interference with general activity	3.9 (3.4)	4.1 (3.5)	0.48	0.49	0.37
	PEG scale score^d^	4.1 (3.1)	4.3 (3.2)	0.58	0.58 (0.49 to 0.66)	
	*GAD-7 anxiety*					
3	Worrying too much about different things	0.7 (1.0)	0.8 (1.0)	0.31	0.30	0.24
2	Not being able to stop or control worrying	0.5 (0.9)	0.7 (1.0)	0.31	0.27	0.22
6	Becoming easily annoyed or irritable	0.5 (0.7)	0.7 (0.9)	0.30	0.25	0.24
4	Trouble relaxing	0.5 (0.9)	0.6 (0.9)	0.30	0.23	0.23
1	Feeling or appearing nervous, anxious or on edge	0.5 (0.8)	0.6 (0.8)	0.27	0.22	0.22
5	Being so restless that it is hard to sit still	0.3 (0.7)	0.3 (0.7)	0.18	0.16	0.16
7	Feeling or appearing afraid as if something awful might happen	0.3 (0.7)	0.3 (0.7)	0.09	0.07	0.08
	GAD-7 scale score^d^	3.3 (2.0)	3.9 (4.5)	0.31	0.28 (0.16 to 0.39)	
	*PHQ-8 depression*					
5	Feeling tired or having little energy	1.2 (1.1)	1.2 (1.1)	0.33	0.31	0.25
4	Poor appetite or overeating	0.7 (1.0)	0.7 (1.0)	0.34	0.30	0.24
2	Feeling or appearing down, depressed, or hopeless	0.4 (0.8)	0.5 (0.9)	0.31	0.30	0.26
3	Trouble falling asleep, trouble staying asleep, or sleeping too much	0.9 (1.1)	0.9 (1.1)	0.27	0.25	0.21
7	Trouble concentrating on things such as reading the newspaper or watching television	0.4 (0.8)	0.4 (0.8)	0.09	0.15	0.09
6	Indicating that s/he feels bad about self, is a failure, or has let self or family down	0.3 (0.8)	0.4 (0.8)	0.22	0.13	0.14
1	Little interest or little pleasure in doing things	0.6 (0.9)	0.6 (0.9)	0.17	0.11	0.11
8	Moving or speaking so slowly that other people could have noticed. …Or the opposite–being so fidgety or restless that s/he has been moving around a lot more than usual	0.5 (0.9)	0.4 (0.9)	0.08	0.06	0.05
	PHQ-8 scale score^d^	5.0 (4.6)	5.2 (5.0)	0.34	0.25 (0.13–0.36)	

^**a**^Items for each scale are ordered in table from highest to lowest quadratic weighted kappa

^**b**^Most of the Spearman correlations are highly significant (p < .0001) except for items only significant at p < .05 (PHQ-8 items 1, 6; GAD-7 items 1, 5) or those not significant (PHQ-8 items 7, 8; GAD-7 item 7)

^**c**^All weighted kappa values are significant (i.e., 95% CI does not include 0) except for GAD-7 items 5 and 7 and PHQ-8 items 1, 6, 7 and 8 (quadratic and linear)

^**d**^For scale scores, the value under the weighted kappa column is the Intraclass Correlation Coefficient (ICC) with 95% CI, calculated using the absolute agreement version of the ICC and specifying rater (patient vs their caregiver) as a fixed effect

Conversely, agreement regarding anxiety and depression was lower. The total score ICC was only 0.28 for anxiety and 0.25 for depression. In addition, the highest weighted kappa at the item level was 0.31, and the kappa was < 0.20 for 4 of the 8 depression items and 2 of the 7 anxiety items.

### Comparison of concordance for items shared by Symtrak and legacy scales

There are 10 items in common between SymTrak and the legacy scales for which the conceptual content is the same and the item wording is either identical or relatively similar, including 6 PHQ-8 items, 3 GAD-7 items, and 1 PEG item. Table 5 shows that patient-proxy concordance was generally comparable for items measured by two different scales.

**Table 5:** Comparison of Patient-Caregiver Concordance on Items Common to Symtrak and Legacy Scales

		Spearman correlation		Weighted Kappa (quadratic)	
Symtrak Item	Legacy Scale Item	Sym Trak	Legacy Scale	Sym Trak	Legacy Scale
	*PHQ-8*				
Less Interest or less pleasure in doing things	Little interest or pleasure in doing things	.16	.17	.17	.11
Feeling sad, down or depressed	Feeling or appearing down, depressed, or hopeless	.35	.31	.29	.30
Feeling tired or having low energy	Feeling tired or having little energy	.37	.33	.35	.31
Trouble falling asleep or trouble staying asleep	Trouble falling asleep, trouble staying asleep, or sleeping too much	.29	.27	.30	.25
Poor appetite or overeating	Poor appetite or overeating	.34	.34	.33	.30
Trouble concentrating on things	Trouble concentrating on things such as reading the newspaper or watching television	.13	.09	.16	.15
	*GAD-7*				
Feeling nervous or anxious	Feeling or appearing nervous, anxious, or on edge	.22	.27	.29	.22
Worrying too much about different things	Worrying too much about different things	.26	.31	.26	.30
Becoming easily annoyed or irritable	Becoming easily annoyed or irritable	.18	.30	.18	.25
	*PEG*				
Pain interfering with daily activities	Pain interfering with general activity	.44	.48	.42	.49

### Concordance related to symptom severity

**Fig. 1:**
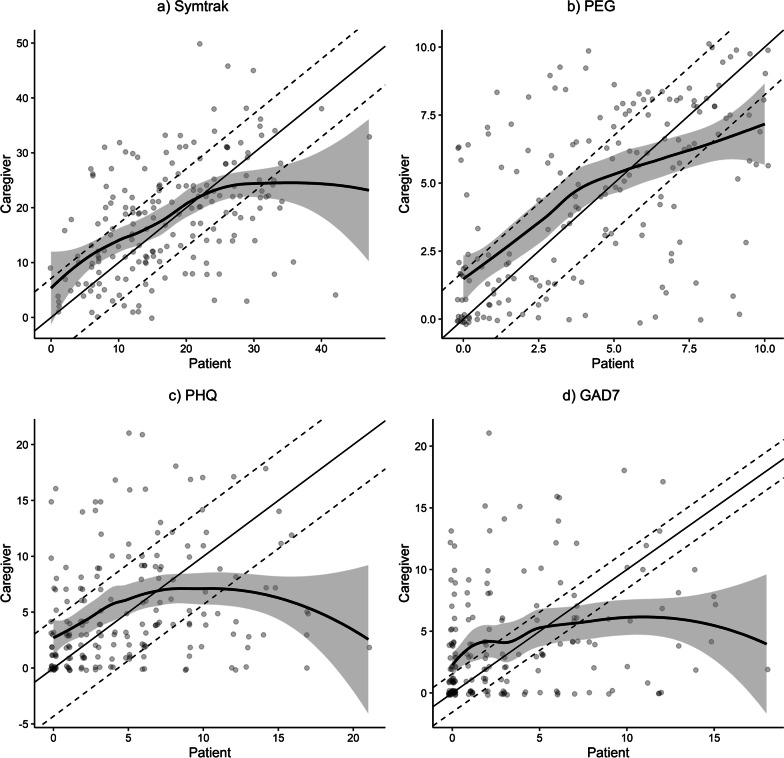
Scatter plot of Caregiver (Proxy) Report versus Patient Self-Report for Patient Symptoms on 4 Scales: **a** SymTrak multimorbidity scale; **b** PEG pain scale; **c** PHQ-8 depression scale; **d** GAD-7 anxiety scale. Higher scores on all 4 scales indicates greater (worse) symptom severity. The solid straight line represents theoretical perfect agreement and the dotted lines are the measurement error bars representing ± 2 SEM (standard error of measurement). The solid curvilinear line represents the fitted actual agreement derived from linear regression and the shaded area represents the confidence limits around the actual agreement

Figure 1 displays the scatter plots showing the association between patient- and caregiver-reported scores. For multi-morbidity and pain (Figs. 1a, b), concordance is generally linear and stronger (steeper slope) at lower scores and tends to decrease or plateau at higher scores. In contrast, concordance for the two psychological scores (Figs. 1c, d) is generally weaker and bidirectional, with a positive slope at lower scores and a flat or slightly negative slope at higher scores. For all 4 scales, most caregiver reports outside the 2-SEM concordance bars exceed patient reports at lower scores and are less than patient reports at higher scores. Additionally, the underestimate by proxies at higher scores is greater for psychological symptoms.

### Predictors of discordance

Table 6 summarizes the results of multinomial logistic regression modeling conducted to explore patient and caregiver characteristics associated with discordance defined as caregiver scores > 2 SEM higher or lower than patient scores (i.e., caregiver overreporting or underreporting, respectively). Discordance was common for the 4 scales, ranging from 33.7% for PHQ-8 depression to 62.4% for GAD-7 anxiety. The severity of patient-reported scores and caregiver task difficulty were associated with discordance. Specifically, higher (worse) patient-reported scale scores were associated with caregiver underreporting and higher caregiver task difficulty was associated with overreporting. Each 1-point increase in the patient-reported scale score increased caregiver underreporting by an OR of 1.17 to 1.60 across the 4 scales, and each 1-point increase in the Oberst caregiver difficulty score increased caregiver overreporting by an OR of 1.07 to 1.10. For a few scales (and complementing these findings), higher patient-reported scores decreased overreporting, and higher caregiver task difficulty decreased underreporting. Other patient and caregiver characteristics were not associated with discordance but were retained in the models to control for their potential effects.

**Table 6:** Multinomial Logistic Regression Models: Correlates of Caregiver Overreporting and Underreporting of Patient-Reported Scale Scores (Four models were run–one for each scale score. Each model has a 3-level dependent variable, with concordance being the reference group; the odds ratios (ORs) for over- and under-reporting are relative to the reference group Differences are calculated as (Caregiver-estimated score)‒(Patient-reported score). Concordance was defined as a difference within ± 2 standard errors of measurement (SEM). Overreporting = Difference > 2 SEM higher. Underreporting = Difference > 2 SEM lower. Percentages in column headings are the proportion of caregivers that over- and underreported for each scale.)

Patient/caregiver variable^†^	SymTrak multimorbidity		PEG pain		PHQ-8 depression		GAD-7 anxiety	
	OR (95% CI)		OR (95% CI)		OR (95% CI)		OR (95% CI)	
	Over (21.3%)	Under (20.2%)	Over (24.2%)	Under (18.7%)	Over (18.7%)	Under (15.0%)	Over (34.2%)	Under (26.2%)
Patient-reported scale score	**0.91 (0.85, 0.97)**	**1.17 (1.10, 1.26)**	**0.84 (0.72, 0.97)**	**1.41 (1.18, 1.69)**	0.90 (0.79, 1.04)	**1.60 (1.33, 1.92)**	0.98 (0.84, 1.14)	**1.55 (1.32, 1.81)**
Caregiver difficulty (Oberst)	**1.10 (1.05, 1.16)**	**0.83 (0.72, 0.95)**	**1.07 (1.02, 1.12)**	0.95 (0.88, 1.03)	**1.08 (1.03, 1.13)**	0.92 (0.81, 1.05)	**1.09 (1.04, 1.15)**	0.98 (0.91, 1.07)
Age, ≥ 75 vs < 75	0.76 (0.30, 1.87)	1.42 (0.51, 3.98)	1.07 (0.46, 2.47)	0.97 (0.37, 2.55)	0.67 (0.28, 1.02)	0.85 (0.22, 3.25)	1.43 (0.64, 3.20)	**3.54 (1.17, 10.7)**
Sex, female vs male	1.86 (0.59, 5.88)	0.91 (0.19, 4.37)	2.32 (0.80, 6.72)	2.99 (0.61, 14.6)	1.72 (0.57, 5.19)	0.95 (0.12, 7.29)	1.29 (0.48, 3.45)	1.99 (0.44, 9.01)
Race, black vs white/other	0.64 (0.25, 1.63)	0.81 (0.28, 2.33)	0.83 (0.35, 1.96)	2.15 (0.78, 5.91)	0.98 (0.40, 2.38)	2.28 (0.52, 9.96)	0.63 (0.28, 1.43)	0.35 (0.11, 1.13)
Income (> 30,000 vs. ≤ 30,000 annually)	0.51 (0.16, 1.56)	0.41 (0.12, 1.47)	0.91 (0.34, 2.38)	1.37 (0.44, 4.32)	1.13 (0.41, 3.10)	7.06 (1.12, 44.6)	0.46 (0.18, 1.20)	0.86 (0.27, 2.76)
Cognitive function (TICS, > 30 vs ≤ 30)	1.46 (0.55, 3.86)	1.13 (0.40, 3.15)	0.52 (0.21, 1.28)	0.48 (0.18, 1.28)	1.61 (0.63, 4.13)	2.68 (0.62, 11.6)	0.61 (0.26, 1.42)	0.79 (0.27, 2.32)
Caregiver sex, female vs male	0.52 (0.18, 1.45)	0.58 (0.13, 2.53)	1.53 (0.55, 4.28)	1.46 (0.37, 5.78)	0.74 (0.27, 2.00)	1.04 (0.18, 6.09)	0.38 (0.14, 1.04)	0.82 (0.20, 3.35)
Caregiver relationship, spouse/partner vs all other	0.79 (0.25, 2.53)	2.30 (0.55, 9.71)	0.86 (0.30, 2.52)	2.75 (0.65, 11.7)	1.85 (0.63, 5.42)	0.64 (0.09, 4.56)	0.55 (0.20, 1.49)	1.20 (0.30, 4.85)

^†^All models were adjusted for patient age as well as caregiver sex and relationship. Additionally, we entered variables associated with under- or over-reporting on bivariate analysis at a P < .05 for at least one of the 4 scales. Thus, multinomial models for all 4 scales included the 9 variables shown in this table

Bolded ORs are those that are significant at p < .05 (i.e., 95% CI does not include 0)

## Discussion

Our study has several important findings. First, both patient and caregiver PRO reports had excellent internal consistency and test–retest reliability. Second, caregiver reports tended to approximate patient reports when total scale and item-level scores were averaged at the group level (i.e., with respect to mean scores), whereas there was substantial variability at the level of the individual patient-caregiver dyad, as reflected in scatterplots and ICC/kappa coefficients. Third, higher patient-reported scale scores were associated with caregiver underreporting, whereas higher caregiver task difficulty was associated with overreporting. Fourth, caregiver underestimates of symptom burden at higher levels of severity were more prominent for depression and anxiety than it was for pain and multi-morbidity measures.

Other studies have also shown reasonable agreement when comparing patient versus proxy mean scores but greater differences when comparing individual patient-proxy scores. Indeed, discordance rates ranged from 34 to 62% for the 4 scales in our study. The greater heterogeneity in concordance at the individual level requires greater caution when interpreting proxy reports in the clinical setting. Whereas some studies have found no directionality in dyad differences (i.e., a similar proportion of patient scores are over- and under-estimated by the proxy) [26–28], research has more commonly shown that proxies tend to overestimate patient-reported symptom severity and impairment [5, 6, 8, 29–39]. Unlike our study, previous studies generally did not evaluate how concordance varies with severity, nor did they adequately control for other potentially confounding patient and caregiver characteristics. Our finding that proxies tend to overestimate impairment at lower levels of symptom severity and underestimate at higher levels therefore warrants further study.

Greater discordance for psychological/internal symptoms than more observable domains such as physical functioning and impairment has also been reported in previous studies [5, 26, 29–31, 33–35, 38]. Even among the performance-based domains, proxy and patient reports may diverge more for higher level functioning than basic functioning (e.g., instrumental vs. basic activities of daily living) [6, 40–42].

Proxy psychological distress and caregiving burden may increase discordance between proxy and patient report, most commonly in the direction of worse ratings of patient PROs [7, 29, 34, 42–46]. Similarly, we found greater caregiver task difficulty led to overestimates of patient symptom severity. Whereas the mechanism for the relationship between caregiving burden and discordance has not been delineated, it is conceivable that caregivers overestimate the patient’s symptom severity as a consequence of their own distress or, alternatively, that patients react to their caregiver’s burden by underestimating self-reported severity. Conversely, neither caregiver sex nor relationship with the patient influenced concordance. Although some studies found that proxies who live with the patient tend to have better concordance, their control for other confounders was less complete than in our study [30, 33, 47].

There is a body of salient pediatric literature on comparing proxy reporting (typically by the parent) to child and adolescent self-report. Like the research in adults, several common themes emerge including greater concordance for group-aggregated scores versus individual dyad-level scores; a tendency for parents to overestimate impairment compared to child self-report; better agreement for observable compared to internal domains (i.e., physical compared to psychological); and an adverse influence on agreement by parental distress [27, 43–46, 48–54]. Whereas some of the findings from pediatric studies may be generalizable to proxy reporting in adults, population differences should also be acknowledged. In children, limitations of self-report are more commonly related to developmental than cognitive impairment factors. Moreover, parents may have a greater actual or perceived responsibility in overseeing treatment and monitoring response. Further, factors such as authority, autonomy, and attachment are not identical for parents and caregivers of adults and thereby may influence the salience and interpretation of proxy report.

Some have made the distinction between what proxies believe the patient would report versus what they think the degree of severity or impairment actually is from their own independent perspective as observers or caregivers [55]. Although we presume that in patients with the capacity to report for themselves, patient report is foremost, it is also possible that the perspectives of persons close to the patient may complement rather than substitute for or replace self-report. Although patients should in most instances still have the primary voice in articulating their level of suffering, distress or impairment (hence the term *patient-reported outcomes*), this does not preclude proxies (who know and observe the patient) from having a valuable vantage point that might further inform evaluation and treatment. Whereas information from proxies is essential for patients with impaired capacity to report for themselves (in which case it serves as a necessary substitute for symptom assessment), proxy report may nonetheless augment scores provided by patients able to self-report. Indeed, investigating the agreement between patient and proxy reports represents a comparison of two perspectives rather than a reliability or validity assessment; patients and caregivers are rating different experiences and perspectives (self vs observer) This contrasts with the typical inter-rater reliability setting where raters are independent observers of the same information. Thus, the generally fair to moderate (instead of substantial) agreement observed here is a point of interest but not an adverse reflection on psychometrics of the scales. In the absence of an objective criterion standard for symptoms and other predominantly internally-experienced (i.e., subjective) phenomena, optimal assessment and therapy might triangulate patient, proxy, and provider/professional perspectives [7, 30, 52, 56].

Because our sample only included cognitively intact adults 65 and older, generalizability to individuals less than 65 as well as individuals with mild to moderate cognitive impairment needs to be further investigated. Moreover, disease progression and functional decline that occur with aging may result in response shift whereby coping, social comparison and other psychological accommodations attenuate the self-evaluation of symptom severity and impairment relative to proxy report [57]. Also, only a few studies have triangulated proxy and patient report with clinician ratings of PROs or performance-based measures relevant to some domains [7, 30, 32, 56, 58]. It would be interesting to do so with the domains assessed by our PAD scales and SymTrak. Additionally, only recently have longitudinal studies compared whether patient and proxy PRO report are similarly responsive over time [59, 60]. This sensitivity to change would be useful to demonstrate for the measures and domains evaluated in our study. Strengths of our study include the size of our sample as well as its racial, educational and income diversity.

In conclusion, proxy PRO reports may be a reasonable alternative in clinical research when patient self-report cannot be obtained and when group mean scores are averaged across individuals. However, the mean scale scores in our sample were relatively low; it is possible that in clinical populations with more severe symptoms or impairment, proxy mean scores may be a less accurate surrogate for patient self-report due to proxy underreporting at the higher score range of scales. In practice, the clinician should realize that proxies tend to underestimate at the higher range of patient-reported depression and anxiety, and this should be considered when making treatment decisions. When both proxy and patient reports are available and clinically significant discordance exists, reconciling the dual perspectives may be preferable to an either-or approach (i.e., one perspective is wrong and the other one is right).

## Data Availability

Data not published within the article are available and will be shared by reasonable request.

## References

[1] Snyder CF, Aaronson NK, Chouchair AK, Elliott TE, Greenhalgh J, Halyard MY, Hess R, Miller DM, Reeve BB, Santana M (2012). Implementing patient-reported outcomes assessment in clinical practice: a review of the options and considerations. Qual Life Res.

[2] Kroenke K, Monahan PO, Kean J (2015). Pragmatic characteristics of patient-reported outcome measures are important for use in clinical practice. J Clin Epidemiol.

[3] Basch E (2017). Patient-reported outcomes—harnessing patients’ voices to improve clinical care. N Engl J Med.

[4] Roydhouse JK, Cohen ML, Eshoj HR, Corsini N, Yucel E, Rutherford C, Wac K, Berrocal A, Lanzi A, Nowinski C, Roberts N (2022). The use of proxies and proxy-reported measures: a report of the international society for quality of life research (ISOQOL) proxy task force. Qual Life Res.

[5] Sneeuw KC, Sprangers MA, Aaronson NK (2002). The role of health care providers and significant others in evaluating the quality of life of patients with chronic disease. J Clin Epidemiol.

[6] Oczkowski C, O'Donnell M (2010). Reliability of proxy respondents for patients with stroke: a systematic review. J Stroke Cerebrovasc Dis.

[7] Robertson S, Cooper C, Hoe J, Hamilton O, Stringer A, Livingston G (2017). Proxy rated quality of life of care home residents with dementia: a systematic review. Int Psychogeriatr.

[8] Roydhouse JK, Gutman R, Keating NL, Mor V, Wilson IB (2018). Proxy and patient reports of health-related quality of life in a national cancer survey. Health Qual Life Outcomes.

[9] Kroenke K, Evans E, Weitlauf S, McCalley S, Porter B, Williams T, Baye F, Lourens SG, Matthias MS, Bair MJ (2018). Comprehensive vs. assisted management of mood and pain symptoms (CAMMPS) trial: study design and sample characteristics. Contemp Clin Trials.

[10] Kroenke K, Spitzer RL, Williams JB, Lowe B (2010). The patient health questionnaire somatic, anxiety, and depressive symptom scales: a systematic review. Gen Hosp Psychiatry.

[11] Kroenke K (2018). Pain measurement in research and practice. J Gen Intern Med.

[12] Kroenke K (2021). The PHQ-9: global uptake of a depression scale. World Psychiatry.

[13] Working Group on Health Outcomes for Older Persons with Multiple Chronic Conditions (2012). Universal health outcome measures for older persons with multiple chronic conditions. J Am Geriatr Soc.

[14] Monahan PO, Kroenke K, Callahan CM, Bakas T, Harrawood A, Lofton P, Frye D, Draucker C, Stump T, Saliba D, Galvin JE, Keegan A, Austrom MG, Boustani M (2019). Development and feasibility of SymTrak, a multi-domain tool for monitoring symptoms of older adults in primary care. J Gen Intern Med.

[15] Monahan PO, Kroenke K, Callahan CM, Bakas T, Harrawood A, Lofton P, Frye D, Draucker C, Stump T, Saliba D, Galvin JE, Keegan A, Austrom MG, Boustani M (2019). Reliability and validity of SymTrak, a multi-domain tool for monitoring symptoms of older adults with multiple chronic conditions. J Gen Intern Med.

[16] Kean J, Monahan PO, Kroenke K, Wu J, Yu Z, Stump TE, Krebs EE (2016). Comparative responsiveness of the PROMIS pain interference short forms, brief pain inventory, PEG, and SF-36 bodily pain subscale. Med Care.

[17] Chen CX, Kroenke K, Stump T, Kean J, Krebs EE, Bair MJ, Damush T, Monahan PO (2019). Comparative responsiveness of the PROMIS pain interference short forms with legacy pain measures: results from three randomized clinical trials. J Pain.

[18] Wu Y, Levis B, Riehm KE, Saadat N, Levis AW, Azar M, Rice DB, Boruff J, Cuijpers P, Gilbody S, Ioannidis JPA, Kloda LA, McMillan D, Patten SB, Shrier I, Ziegelstein RC, Akena DH, Arroll B, Ayalon L, Baradaran HR, Baron M (2020). Equivalency of the diagnostic accuracy of the PHQ-8 and PHQ-9: a systematic review and individual participant data meta-analysis. Psychol Med.

[19] Kroenke K, Spitzer RL, Williams JBW, Monahan PO, Lowe B (2007). Anxiety disorders in primary care: prevalence, impairment, comorbidity, and detection. Ann Intern Med.

[20] Stein MB, Craske MG (2017). Treating anxiety in 2017: optimizing care to improve outcomes. JAMA.

[21] Bakas T, Austin JK, Jessup SL, Williams LS, Oberst MT (2004). Time and difficulty of tasks provided by family caregivers of stroke survivors. J Neurosci Nurs.

[22] Sim J, Wright CC (2005). The kappa statistic in reliability studies: use, interpretation, and sample size requirements. Phys Ther.

[23] Fleiss JL, Cohen J (1973). The equivalence of weighted kappa and the intraclass correlation coefficient as measures of reliability. Educ Psychol Measur.

[24] Landis JR, Koch GG (1977). The measurement of observer agreement for categorical data. Biometrics.

[25] Chen CX, Kroenke K, Stump T, Kean J, Carpenter JS, Krebs EE, Bair MJ, Damush TM, Monahan PO (2018). Estimating minimally important differences for the PROMIS pain interference scales: results from three randomized clinical trials. Pain.

[26] Gabbe BJ, Lyons RA, Sutherland AM, Hart MJ, Cameron PA (2012). Level of agreement between patient and proxy responses to the EQ-5D health questionnaire 12 months after injury. J Trauma Acute Care Surg.

[27] Lifland BE, Mangione-Smith R, Palermo TM, Rabbitts JA (2018). Agreement between parent proxy report and child self-report of pain intensity and health-related quality of life after surgery. Acad Pediatr.

[28] Scott EL, Foxen-Craft E, Caird M, Philliben R, deSebour T, Currier E, Voepel-Lewis T (2020). Parental proxy promis pain interference scores are only modestly concordant with their child’s scores: an effect of child catastrophizing. Clin J Pain.

[29] Rothman ML, Hedrick SC, Bulcroft KA, Hickam DH, Rubenstein LZ (1991). The validity of proxy-generated scores as measures of patient health status. Med Care.

[30] Sprangers MA, Aaronson NK (1992). The role of health care providers and significant others in evaluating the quality of life of patients with chronic disease: a review. J Clin Epidemiol.

[31] Magaziner J, Bassett SS, Hebel JR, Gruber-Baldini A (1996). Use of proxies to measure health and functional status in epidemiologic studies of community-dwelling women aged 65 years and older. Am J Epidemiol.

[32] Ball AE, Russell EM, Seymour DG, Primrose WR, Garratt AM (2001). Problems in using health survey questionnaires in older patients with physical disabilities. Can proxies be used to complete the SF-36?. Gerontology.

[33] Yip JY, Wilber KH, Myrtle RC, Grazman DN (2001). Comparison of older adult subject and proxy responses on the SF-36 health-related quality of life instrument. Aging Ment Health.

[34] Williams LS, Bakas T, Brizendine E, Plue L, Tu W, Hendrie H, Kroenke K (2006). How valid are family proxy assessments of stroke patients’ health-related quality of life?. Stroke.

[35] Carod-Artal FJ, Ferreira Coral L, Stieven Trizotto D, Menezes Moreira C (2009). Self- and proxy-report agreement on the Stroke Impact Scale. Stroke.

[36] Jensen-Dahm C, Vogel A, Waldorff FB, Waldemar G (2012). Discrepancy between self- and proxy-rated pain in Alzheimer’s disease: results from the Danish Alzheimer intervention study. J Am Geriatr Soc.

[37] Hack TF, McClement SE, Chochinov HM, Dufault B, Johnston W, Enns MW, Thompson GN, Harlos M, Damant RW, Ramsey CD, Davison SN, Zacharias J, Strang D, Campbell-Enns HJ (2018). Assessing symptoms, concerns, and quality of life in noncancer patients at end of life: how concordant are patients and family proxy members?. J Pain Symptom Manag.

[38] Alvarez-Nebreda ML, Heng M, Rosner B, McTague M, Javedan H, Harris MB, Weaver MJ (2019). Reliability of proxy-reported patient-reported outcomes measurement information system physical function and pain interference responses for elderly patients with musculoskeletal injury. J Am Acad Orthop Surg.

[39] von Essen L (2004). Proxy ratings of patient quality of life–factors related to patient-proxy agreement. Acta Oncol.

[40] Magaziner J, Zimmerman SI, Gruber-Baldini AL, Hebel JR, Fox KM (1997). Proxy reporting in five areas of functional status. Comparison with self-reports and observations of performance. Am J Epidemiol.

[41] Ostbye T, Tyas S, McDowell I, Koval J (1997). Reported activities of daily living: agreement between elderly subjects with and without dementia and their caregivers. Age Ageing.

[42] Long K, Sudha S, Mutran EJ (1998). Elder-proxy agreement concerning the functional status and medical history of the older person: the impact of caregiver burden and depressive symptomatology. J Am Geriatr Soc.

[43] Eiser C, Varni JW (2013). Health-related quality of life and symptom reporting: similarities and differences between children and their parents. Eur J Pediatr.

[44] Abate C, Lippe S, Bertout L, Drouin S, Krajinovic M, Rondeau E, Sinnett D, Laverdiere C, Sultan S (2018). Could we use parent report as a valid proxy of child report on anxiety, depression, and distress? A systematic investigation of father-mother-child triads in children successfully treated for leukemia. Pediatr Blood Cancer.

[45] Oltean II, Ferro MA (2019). Agreement of child and parent-proxy reported health-related quality of life in children with mental disorder. Qual Life Res.

[46] Mack JW, McFatrich M, Withycombe JS, Maurer SH, Jacobs SS, Lin L, Lucas NR, Baker JN, Mann CM, Sung L, Tomlinson D, Hinds PS, Reeve BB (2020). Agreement between child self-report and caregiver-proxy report for symptoms and functioning of children undergoing cancer treatment. JAMA Pediatr.

[47] Bassett SS, Magaziner J, Hebel JR (1990). Reliability of proxy response on mental health indices for aged, community-dwelling women. Psychol Aging.

[48] Upton P, Lawford J, Eiser C (2008). Parent-child agreement across child health-related quality of life instruments: a review of the literature. Qual Life Res.

[49] Cohen LL, Vowles KE, Eccleston C (2010). Adolescent chronic pain-related functioning: concordance and discordance of mother-proxy and self-report ratings. Eur J Pain.

[50] Lal SD, McDonagh J, Baildam E, Wedderburn LR, Gardner-Medwin J, Foster HE, Chieng A, Davidson J, Adib N, Thomson W, Hyrich KL (2011). Agreement between proxy and adolescent assessment of disability, pain, and well-being in juvenile idiopathic arthritis. J Pediatr.

[51] Hermont AP, Scarpelli AC, Paiva SM, Auad SM, Pordeus IA (2015). Anxiety and worry when coping with cancer treatment: agreement between patient and proxy responses. Qual Life Res.

[52] Galloway H, Newman E (2017). Is there a difference between child self-ratings and parent proxy-ratings of the quality of life of children with a diagnosis of attention-deficit hyperactivity disorder (ADHD)? A systematic review of the literature. Atten Defic Hyperact Disord.

[53] Alcantara J, Ohm J, Alcantara J (2017). Comparison of pediatric self reports and parent proxy reports utilizing PROMIS: results from a chiropractic practice-based research network. Complement Ther Clin Pract.

[54] Birnie KA, Richardson PA, Rajagopalan AV, Bhandari RP (2020). Factors related to agreement between child and caregiver report of child functioning with chronic pain: PROMIS pediatric and parent proxy report. Clin J Pain.

[55] Pickard AS, Knight SJ (2005). Proxy evaluation of health-related quality of life: a conceptual framework for understanding multiple proxy perspectives. Med Care.

[56] Pepin V, Alexander JL, Phillips WT (2004). Physical function assessment in cardiac rehabilitation: self-report, proxy-report and performance-based measures. J Cardiopulm Rehabil.

[57] Vanier A, Oort FJ, McClimans L, Ow N, Gulek BG, Bohnke JR, Sprangers M, Sebille V, Mayo N (2021). Response shift in patient-reported outcomes: definition, theory, and a revised model. Qual Life Res.

[58] Prichard RA, Zhao FL, McDonagh J, Goodall S, Davidson PM, Newton PJ, Farr-Wharton B, Hayward CS (2021). Discrepancies between proxy estimates and patient reported, health related, quality of life: minding the gap between patient and clinician perceptions in heart failure. Qual Life Res.

[59] Lapin BR, Thompson NR, Schuster A, Honomichl R, Katzan IL (2021). The validity of proxy responses on patient-reported outcome measures: are proxies a reliable alternative to stroke patients’ self-report?. Qual Life Res.

[60] Wolf RT, Ratcliffe J, Chen G, Jeppesen P (2021). The longitudinal validity of proxy-reported CHU9D. Qual Life Res.

